# The Effect of Solid-State Processes and Heat Treatment on the Properties of AA7075 Aluminum Waste Recycling Nanocomposite

**DOI:** 10.3390/ma14216667

**Published:** 2021-11-05

**Authors:** Huda Mohammed Sabbar, Zulkiflle Leman, Shazarel Shamsudin, Suraya Mohd Tahir, Che Nor Aiza Jaafar, Azmah Hanim Mohamed Ariff, Nur Ismarrubie Zahari, Mohammed H. Rady

**Affiliations:** 1 Department of Mechanical and Manufacturing Engineering, Faculty of Engineering, Universiti Putra Malaysia, Serdang 43400, Malaysia; su_mtahir@upm.edu.my (S.M.T.); cnaiza@upm.edu.my (C.N.A.J.); azmah@upm.edu.my (A.H.M.A.); rubie@upm.edu.my (N.I.Z.); 2 Advanced Engineering Materials and Composites Research Centre, Faculty of Engineering, Universiti Putra Malaysia, Serdang 43400, Malaysia; 3 Laboratory of Biocomposite Technology, Institute of Tropical Forestry and Forest Products, Universiti Putra Malaysia, Serdang 43400, Malaysia; 4 Sustainable Manufacturing and Recycling Technology, Advanced Manufacturing and Materials Centre (SMART-AMMC), Universiti Tun Hussein Onn Malaysia, Batu Pahat 86400, Malaysia; shazarel@uthm.edu.my; 5 College of Engineering, Wasit University, Kut 52001, Iraq; mradhi@uowasit.edu.iq

**Keywords:** aluminium alloy AA7075, ECAP, heat treatment, hot extrusion, ZrO_2_

## Abstract

Direct solid-states, such as hot extrusion and equal channel angular pressing (ECAP), are alternative and efficient solid-state processes for use in recycling aluminium scrap. These processes utilise less energy and are eco-friendly. Ceramic particles such as ZrO_2_ are suggested as alternatives in the production of metal composites. This study investigated and optimised the effects of various parameters of reinforced ZrO_2_ nanoparticles on the mechanical and physical properties via response surface methodology (RSM). These parameters were the volume fraction (VF), preheating temperature (T), and preheating time (t). The effects of these parameters were examined before and after the heat treatment condition and ECAP. Each parameter was evaluated at varying magnitudes, i.e., 450, 500, and 550 °C for T, 1, 2, and 3 h for t, and 1, 3, and 5% for VF. The effect that process variables had on responses was elucidated using the factorial design with centre point analysis. T and VF were crucial for attaining the optimum ultimate tensile strength (UTS) and microhardness. Reducing VF increased the mechanical properties to 1 vol% of oxide. The maximum hardness of 95 HV was attained at 550 °C, 1.6 h, and 1 vol% ZrO_2_ with a density of 2.85 g/cm^3^ and tensile strength of 487 MPa. UTS, density, and microhardness were enhanced by 14%, 1%, and 9.5%, respectively. Additionally, the hot extrusion parameters and ECAP followed by heat treatment strengthened the microhardness by 64% and density by 3%. Compression pressure and extrusion stress produced in these stages were sufficient to eliminate voids that increased the mechanical properties.

## 1. Introduction

Conventionally, the recycling of aluminium scraps is carried out through re-melting at high temperatures with the recovery of most of the materials [1]. However, the loss of materials and the high-energy requirement in the conventional recycling method [2] have encouraged the development of eco-friendly and economically viable methods to address the environmental issues of air pollution. This method, broadly known as solid-state recycling, involves the direct treatment of alloy chips [3,4]. Solid-state recycling, such as hot extrusion and ECAP, optimises energy, using the plastic deformation technique to recycle metal scraps and various alloys [5,6,7]. Furthermore, heat treatment after solid-state recycling, i.e., hot extrusion, improves the mechanical properties of the alloy with the formation of secondary phases and the homogeneous distribution of fine precipitates [6]. The hot extrusion solid-state recycling process not only conserves the environment but also prevents the generation of new waste material [7].

Meanwhile, the technique of ECAP is used to improve the effect of reinforcement on the properties of light metals and alloys of aluminium, copper, and titanium. The ECAP technique produces composite materials with high mechanical properties to enhance the properties of billets. In general, studies on ECAP focused on metallic alloys, pure metals, and plastic deformation [8]. For example, processing metal matrix composite (MMC) materials through extrusion or ECAP has been widely used to manufacture ultrafine-grained structures for various engineering materials.

This research study uses extrusion along with ECAP to recycle metal matrix composites (MMCs). The most common SPD technique is ECAP, which uses the ECAP die with different angles [9]. Apparently, the ECAP technique is flexible and has been deployed in combination with hot extrusion. This informed the choice of ECAP in combination with hot extrusion in the current study.

The physical and mechanical properties and microstructure of products extruded using the solid-state recycling of aluminium alloy chips are dependent on the number of hot extrusion parameters [10]. Temperature-related parameters, the extrusion ratio, die geometry, chip morphology, and ram speed are relevant factors that need to be well regulated to obtain qualitative products from the recycling process [11,12].

This research intends to propose a new approach to improve the performance of aluminium composites made of chips with the addition of ZrO_2_ particles. Moreover, the chip-based composite-reinforced ZrO_2_ contents offer alternative sources to manufacturing automotive industries to recycle, reuse the machined materials as a secondary source of metal, and protect our earth from greenhouse gas for a sustainable life. This study focused on examining the effects of preheating time (t), preheating temperature (T), and volume fraction (VF) on the mechanical and physical properties of a ZrO_2_ aluminium chips nanocomposite. This nanocomposite was produced through the hot extrusion method followed by ECAP to compare the result with heat treatment. The influence of each factor was analysed using the factorial design, followed by RSM. The microstructure and the average grain sizes of the extrudates were also investigated.

## 2. Materials and Methods

### Fabrication of Hybrid Aluminium Nanocomposite

Samples of recycled MMC chips were fabricated with the addition of AA7075 aluminium chips and ZrO_2_ particles to enhance the mechanical and physical properties of the alloy. ZrO_2_ nanoparticles were added due to their robust mechanical and electrical properties, good wear resistance and corrosion resistance, and a wide bandgap. The sizes of the AA7075 chips (Table 1) were verified using a digital venire calliper. Table 2 shows the chemical composition of the AA7075 samples.

In the fabrication, the AA7075 aluminium alloy chips were snipped using a computer numerical control (CNC) machine with a depth cut of 1 mm. Following the standard method of the American Society for Testing and Materials (ASTM) G131-96, the chips were degreased with acetone in an ultrasonic bath for 30 min to remove the contaminants of the coolant oil and other substances. The chips were dried at 80 °C for 30 min thenmixed separately with 1%, 3%, and 5% ZrO_2_ nanoparticles using a three-dimensional (3D) mixer (SYH-15), China. for 2 h at the speed of 35 rpm. Billets were formed using cold compaction in a cylindrical die with a diameter of 30 mm and a length of 100 mm with a pressing force of 50 kN.

**Figure 1:**
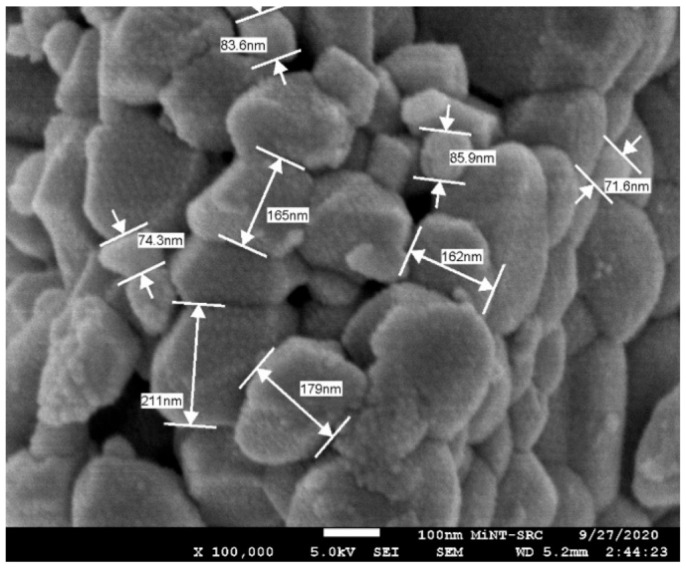
ZrO_2_ nanoparticle size and shape.

**Figure 2:**
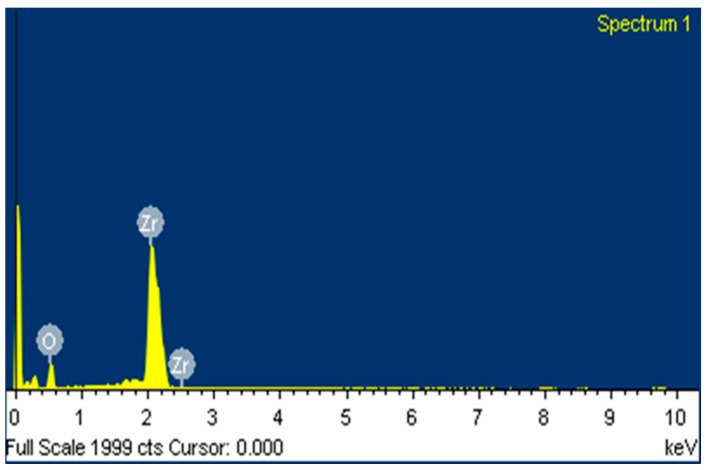
ZrO_2_ nanoparticle EDX.

FESEM-EDX were used in the study of the morphology of ZrO_2_ particle (Suzhou Beike Technology Co., Suzhou, China) shape and size with the magnification of ×100,000 (Figure 1 shows the particle sizes of ZrO_2_, ranging from 70 to 211 nm). They deviated slightly from previous findings due to the inefficient dispersion of the powder during particle size analysis in this study. The purity of the reinforcement (Table 3) shows the EDX analysis (Figure 2) of the ZrO_2_ nanopowder, confirming the presence of Zr and O.

Minitab 18 software was used to perform RSM. The experimental design of the research was performed in the full factorial design, where the three main parameters were 1–5% vol ZrO_2_-reinforced nanoparticle, a 1–3 h time period and a temperature of 450–550 °C; these processing values were applied. In this work, the experiment’s design was intended to help develop the optimisation and proposal of MMC composites. The best overall optimum parameters, such as particles and processing temperature, were investigated. Then, the RSM model Equation (1) was suggested as the sufficiency, and the linear model was capable of defining the relationship between the response or other process factors in the process.

(1) $$y=b_{0}+b_{1}X_{1}+b_{2}X_{2}+\cdots +b_{k}X_{k}$$

In the regression equation, *y* is the response variable, *b*_0_ is the constant, *b*_1_, *b*_2_, …, *b_k_* are the coefficients, and *X*_1_, *X*_2_, …, *X_k_* are the values of the terms.

Parameters such as preheating temperatures, compositions of the aluminium chip, and ZrO_2_ reinforcement materials are shown in Table 4.

In hot extrusion (the A process), the billet was preheated in a container with a ceramic heater to facilitate plastic deformation (Table 5) [13]. A graphite-based lubricant was used in the inner die surface and container in every extrusion cycle to prevent the increase in load in the extrusion due to friction [3].

**Figure 3:**
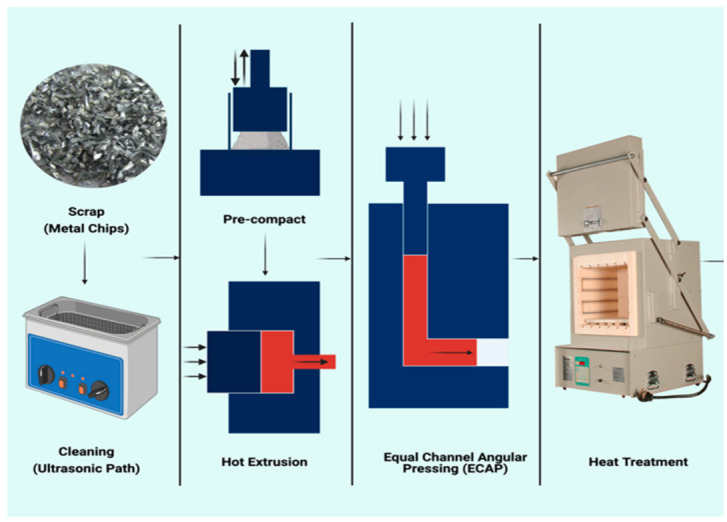
The sequence of the chip pre-processing before and after consolidation [14].

After hot extrusion, the final products were divided into 28 specimens; then, the optimal properties were sample treated with hot ECAP (the B process) that used a cold press hydraulic machine of 500 kN, an ECAP die, heaters, and thermocouples, and the ECAP die consisted of two parts, with a channel of 12 mm × 12 mm with an inner angle of 90° and an outer angle of 20°. Temperatures were measured using a K-type thermocouple with a diameter of 3 mm. Figure 3 shows the details of the setup.

**Figure 4:**
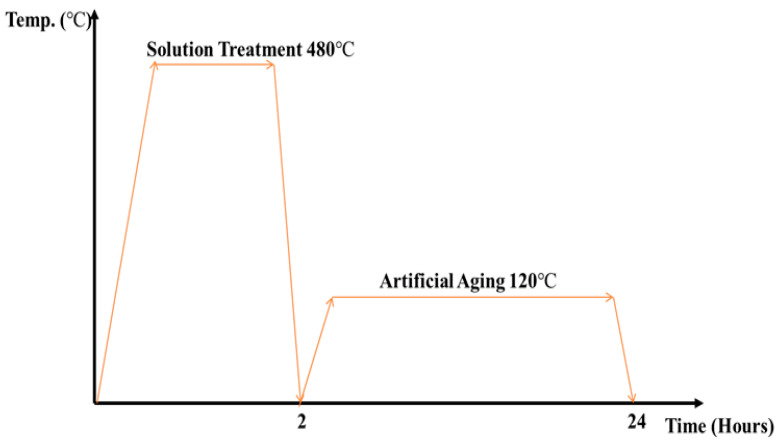
Heat treatment process.

Heat treatment (HT) was performed using an electrical box furnace at a quenching temperature of 465 °C for 55 min and an artificial aging process at 120 °C for 24 h (Figure 4). Water was used as the quenching medium.

The samples extruded from the hot extrusion die underwent tensile and microhardness tests. For tensile testing, the extruded samples were snipped according to the ASTM E8-E8M standard for producing dog-bone-shaped samples. The tensile test with an initial strain rate of 2.53 × 10^−3^ s^–1^ was performed at room temperature until failure. Based on the ASTM E92-82 standards, the microhardness test was performed using a Micro Vikers Hardness tester (Shimadzu) with a Knoop indenter, 25 g load (0.9807 N), indent time of 15 s, and a holding time of 10 s at room temperature following the grinding of the surfaces for each sample for a balanced indentation. Samples were tested using a square-based, pyramid-shaped diamond indenter, indenting the samples in three places (top, centre, and bottom). These three values were averaged to yield the hardness for each sample. Microhardness was dependent on the plasticity, elasticity, strength, and ductility of the samples.

The reinforced particles distribution, chip boundaries, and the grain size of the samples were evaluated using atomic force microscopy (AFM) and Scanning Electron Microscopy (SEM). Specifically, AFM was used to test the conductive materials by investigating the roughness of surface topography from the micro- to nanoscales of the prepared samples [15].The morphology of ZrO_2_ (shape and size) was evaluated using Field Emission Scanning Electron Microscopy with Energy Dispersive X-Ray Spectroscopy (FESEM-EDX) in three magnifications, i.e., 100×, 300×, and 500×. Meanwhile, the density was evaluated using Archimedes’ water immersion principle with samples in circular pieces of approximately 1 mm in diameter and thickness and with the help of the HR-250AZ-Compact Analytical Balance density determination kit. Specimens were weighed in the air and distilled water to record the weight in different environments. Each sample was immersed in distilled water during the density measurement at room temperature. The density of the composite material was calculated using Equation (2) below [16].

(2) $$\mathrm{Bulk}\;\mathrm{density},\;\rho_{b}=\frac{A}{\left|B\right|}\times \;\mathrm{density}\;\mathrm{of}\;\mathrm{distilled}\;\mathrm{water}$$

where *A* = weight in the air and *B* = weight in liquid. The difference of theoretical and measured density values gave the percentage of pores. 

## 3. Results and Discussion

### 3.1. Tensile Strength

**Figure 5:**
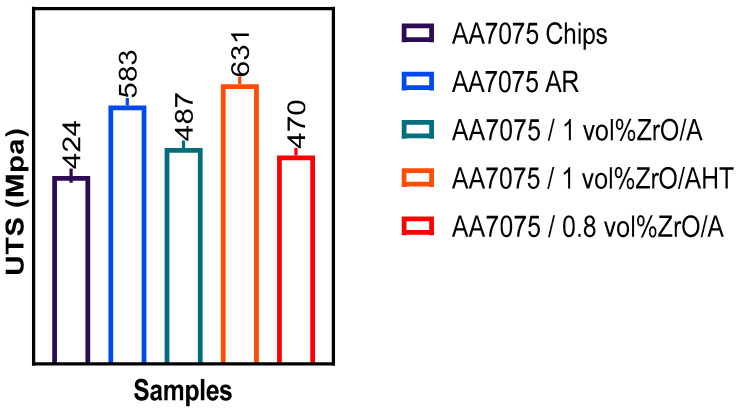
UTS of various composite samples.

The tensile strength of the Al chips and Al-ZrO_2_ composites produced from hot extrusion was congruent with the original material of T6-AA7075 containing 1% ZrO_2_ nanoparticles and a preheating temperature of 550 °C. Figure 5 shows the ultimate tensile strength (UTS) of the extruded samples due to work hardening. The tensile strength values were 583 MPa for the sample AA7075 as received (AR), 470 MPa for the sample after hot extrusion with 0.8% ZrO_2_, and 631 MPa after heat treatment. The extrusion encompassed the recycled Al chips and Al-1 vol% ZrO_2_ composites with a tensile strength of 424 MPa and 487 MPa, respectively, showing an enhancement in the mechanical properties of MMC. The tensile strength of Al–ZrO_2_ was enhanced with the addition of nanoparticles until 1 vol%, after which it decreased with the increment of nanopowder and optimised processing parameters for hot extrusion. The strength of the recycled MMC-based composites increased due to work hardening. These results showed a substantial increase in properties in heat-treated samples. 

**Figure 6:**
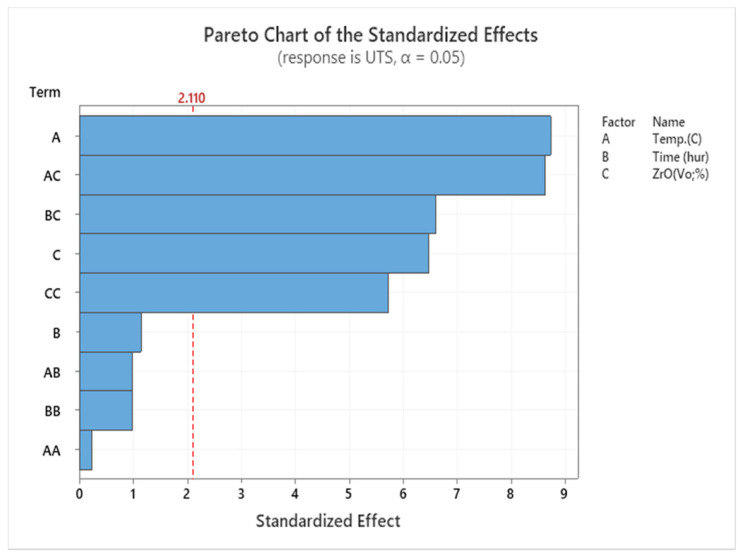
Pareto chart for UTS.

The results suggest that specimens heated to the maximum temperature may have greater tensile strength. As the temperature rises, AMC becomes stronger and exposes the enhanced microstructure. By increasing the temperature to 550 °C and reducing the volume fraction to 1%, the tensile strength is increased to its maximum value. According to RSM’s explanation, it is obvious that the key parameter list that affects the investigation factors of the UTS of the extruded sample is T and ZrO_2_. On the contrary, time is not significant. SEM shows the sources of variance in Table 6, showing the *p*-value of the linear model. Temperature and reinforcement are significant, but the lack of fit is not significant. In the factor list, these factors are represented by *p* < 0.05, as shown in the Pareto charts shown in Figure 6. 

The coefficient of determination, R^2^, adjusted R^2^ and predicted R^2^ are other criteria used to evaluate the adequacy of the model. For UTS, the value of R^2^, R^2^-adjusted and R^2^-predicted are 94.59%, 91.41%, and 85.27%, respectively. The value of R^2^ indicates that about 5% of the total variation was not explained by the model. This implies that the regression model provides an excellent explanation of the relationship between the independent variables and the response. By implication, the regression model is good and supports the experimental observations. Therefore, the result proves that the zirconium oxide nanoparticles added into the recycled AA7075 chips enhance the tensile stress of the newly developed composite material.

**Figure 7:**
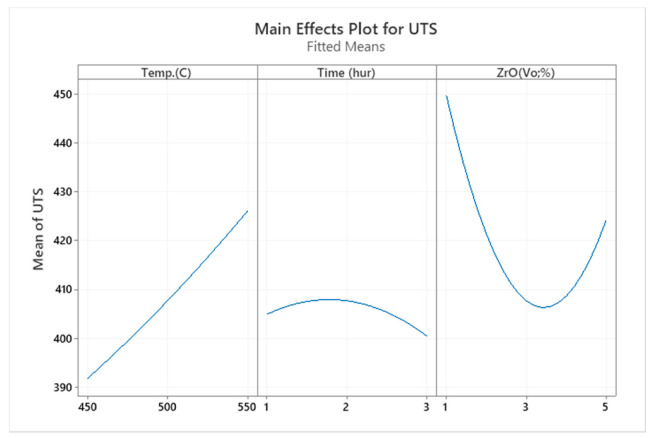
The main effect plot for UTS.

**Figure 8:**
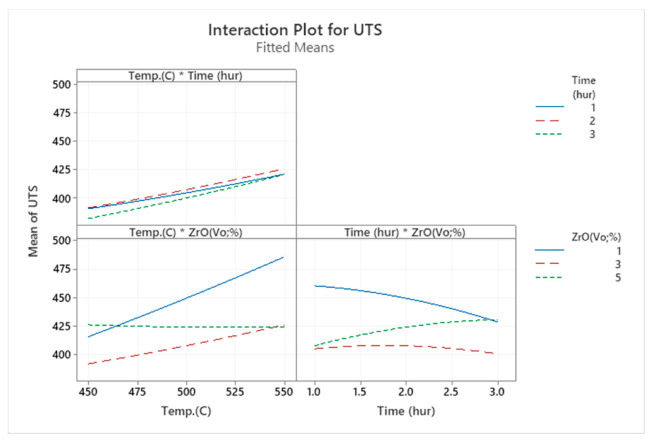
The interaction plot for UTS.

Specimens heated to the maximum temperature appeared to have greater tensile strength. As the temperature rose, aluminium matrix composite (AMC) became stronger and exposed the enhanced microstructure. Figure 5 shows that at 550 °C with the volume fraction reduced to 1%, the tensile strength attained its maximum value, i.e., 487 MPa. Additionally, the samples extruded at 450 °C with a 5% ZrO_2_ volume fraction showed poor strength (426 MPa). These results were consistent with that of another study [13]. Figure 7 shows the main effect plot in the full factorial analysis, indicating that all the centre points, from low to high preheating temperature settings, were extremely close to the straight line of the average tensile strength. Preheating temperature (T) as a factor had a linear relationship to the UTS response. The tensile stress tended to decrease considerably with the increase in zirconium oxide up to 3% vol, where it started rise. Thus, the maximum UTS was obtained at the peak temperature at 550 °C and volume fraction at 1%, as shown in the interaction plots in Figure 8.

**Figure 9:**
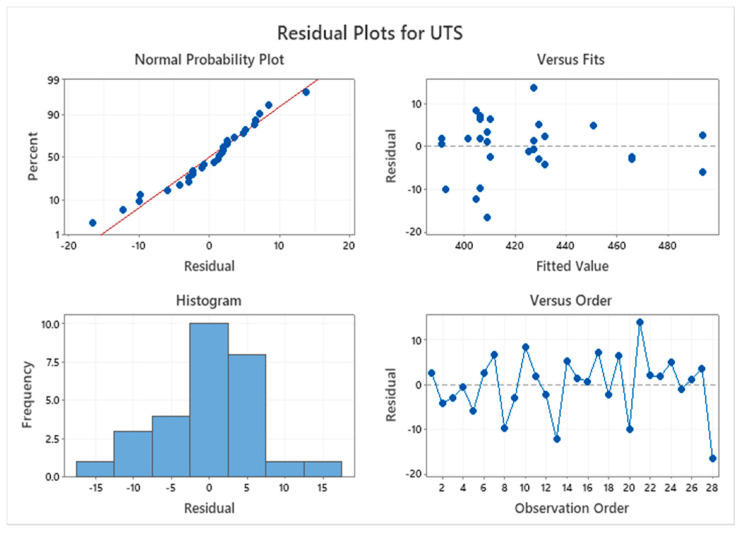
The residual plot for UTS.

Figure 9 shows the residual plot for tensile strength. It is observed that the residual for UTS almost displays curvature in the normal probability plot. The closeness of the graph indicates that errors are negligible since they are in the tolerable margin.

### 3.2. Effects of ZrO_2_ Nanoparticles on Microhardness

**Figure 10:**
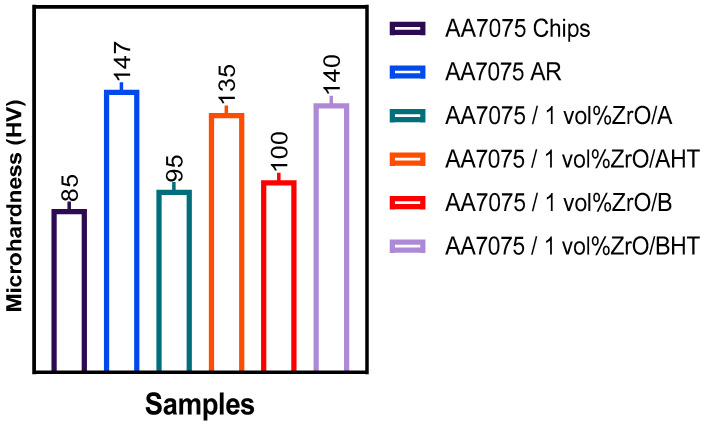
The hardness of the samples.

**Figure 11:**
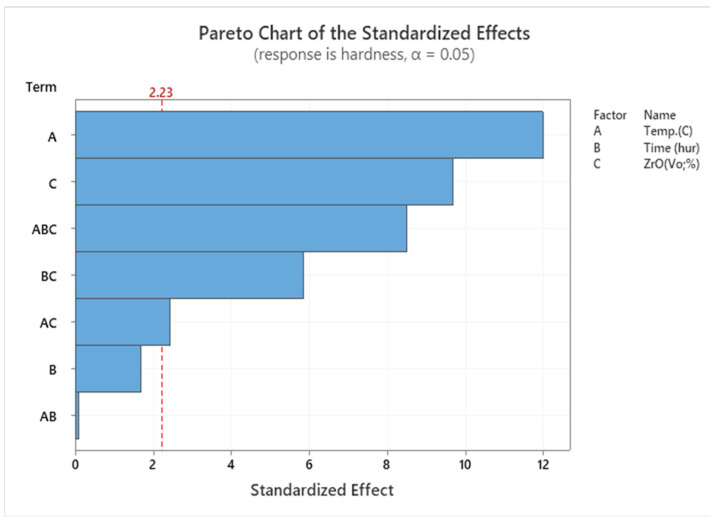
Pareto chart for hardness.

Figure 10 shows the hardness of samples and Figure 11 shows the Pareto chart of the standardised effects. Regarding the hot extruded sample, the hardness (95 HV) was attained at 550 °C, 1 h, and 1% for T, t, and VF had increased to 135 HV after the heat treatment. Subsequent ECAP reduced the microhardness to 100 HV, but it increased again to 140 HV with further heat treatment. 

The full factorial results in Table 7 show that the model is significant. The *p*-value for the model is lower than 0.05 (i.e., α = 0.05, or 95% confidence). This indicates that the model is considered to be statistically significant. The curvature value is 0.18, more than 0.05, which means that is insignificant as desired; hence, the model fits the experimental data and the independent variables have considerable effects on the responses.

For microhardness, the value of R^2^, R^2^-adjusted and R^2^-predicted are 97.25%, 95.05%, and 90%, respectively. The value of R^2^ indicates that less than 3% of the total variation was not explained by the model. This means that the regression model provides an excellent explanation of the relationship between the independent variables and the response. The “Pred R^2^” of 0.9505 is within a reasonable agreement with the “Adj R-Squared” of 0.9.3.3. 

**Figure 12:**
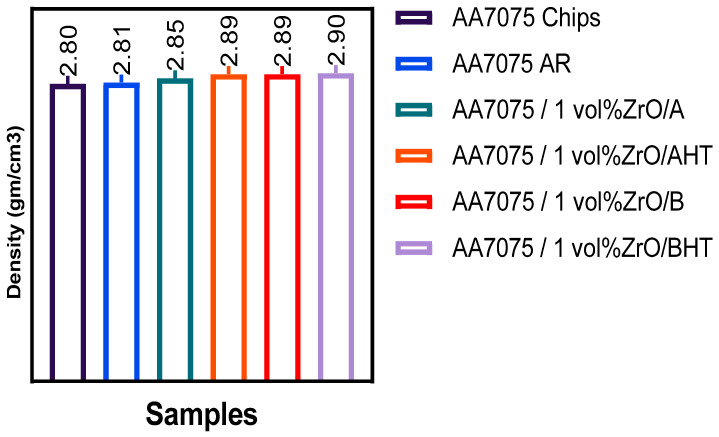
The results of density.

The full factorial conducted for density test (Figure 12) shows the density of the samples. The RSM analysis indicated that the density increased along all the parameters used in sample extrusion. The maximum density (2.89 g/cm^3^) was attained at 450 °C, 3 h, and 5% for T, t, and VF, respectively; for the ECAP sample, the maximum density (2.89 g/cm^3^) was attained at 550 °Ċ, 1.58 h, and 1% for T, t, and VF and 2.9 g/cm^3^ after heat treatment, indicating that high temperature and dense compaction of the chips resulted in poor inter-chip consolidation [17]. These extrusion conditions were only suitable for eliminating the voids and were incapable of improving the chip bonding. Although samples extruded at high temperatures had higher strength [18], such conditions resulted in lower density due to the formation of residual voids and cracks in the extruded products. In general, the preheating temperature along with preheating time and volume fraction of ZrO_2_ were relatively more crucial for determining the density in the hot extrusion of the solid-state recycling method.

The factorial regression results in Table 8 show that the model is significant. The *p*-value for the model is lower than 0.05 (i.e., α = 0.05, or 95% confidence). This indicates that the model is considered to be statistically significant. The value of lack of fit term is 0.126, more than 0.05. The interpretation of this is that the model is significant, as desired. The model fit the experimental data and the independent variables have considerable effects on the response.

The coefficient of determination for density, the value of *R^2^*, *R^2^*-adjusted, and *R^2^*-predicted are 98.24%, 96.83%, and 93.22%, respectively. The value of *R^2^* indicates that only about 1% of the total variation was not explained by the model. This implies that the regression model provides an excellent explanation of the relationship between the independent variables and the response.

**Figure 13:**
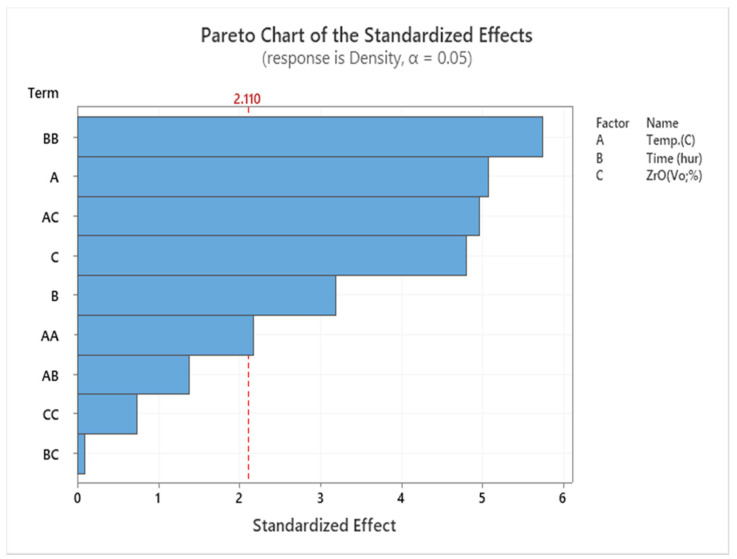
Pareto chart for density.

**Figure 14:**
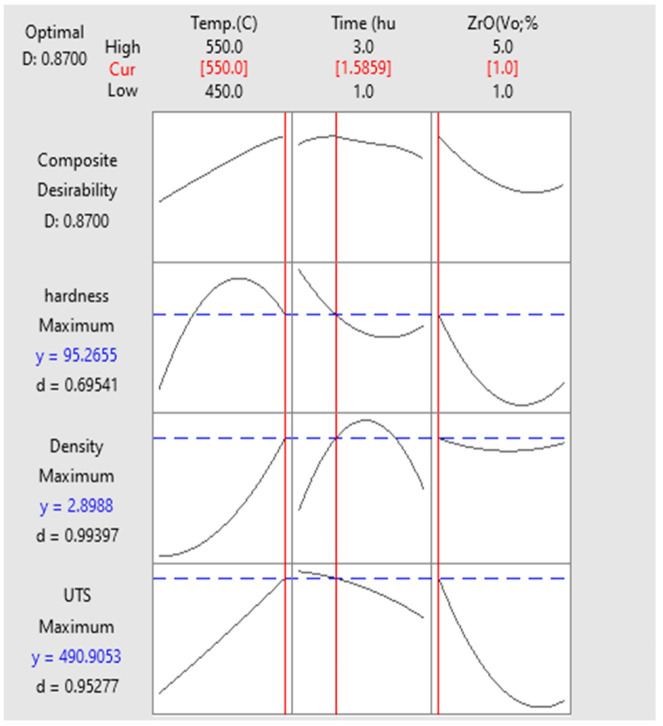
UTS, microhardness and density optimal plot.

The results in Table 8, the Pareto chart (Figure 13), and the comparison of hardness, density, and UTS (Figure 14) clearly show these relationships. 

### 3.3. Multi-Objective Optimisation 

The multi-objective optimisation results of this study were consistent with the above optimal results, especially with T and VF. The maximum responses of this study for T, t, and VF were 550 °C, 1.6 h, and 1 vol% ZrO_2_. The average UTS, microhardness, and density of this study were 490 MPa, 95.2 HV, and 2.89 g/cm^3^, respectively (Figure 14).

### 3.4. Validation Test and Prediction

The results of the experimental tensile strength, microhardness, and density tests for the three specimens are given in Table 9. Three confirmation tests (CTs) were performed to validate the empirical results. Based on the design of experiment (DOE) analysis, the model can be seen in this table; the calculated errors are within manageable limits and small compared to the obtained results. The calculated errors between the experimental and the predicted result (Table 10) are within the range of 0.1% to 10.0% or ±10%. Clearly, these results successfully confirm the reproducibility of the experimental data.

### 3.5. Scanning Electron Microscopy (SEM) Analysis

**Figure 15:**
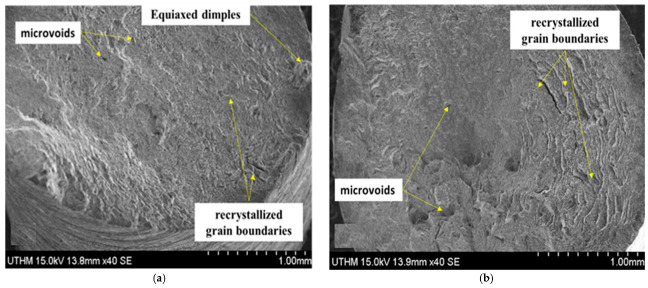
(**a**) AA7075 chips (**b**) 1 vol%ZrO_2_/A.

Surface fracture of the ZrO_2_ nanoparticles and the damage mechanism for the AA7075 extruded sample (Figure 15a), and the microstructure of the reinforced samples with ZrO_2_ nanoparticles (Figure 15b) with a uniform distribution of particles in the MMC are shown. The ceramic phase was dark, and the white one was the metal matrix of AA7075. The distribution of the composite was dependent on the good interfacial bonding between ZrO_2_ nanoparticles and the matrix along the grain boundaries that redacted the equiaxed dimples. However, microvoids were visible in some regions. 

### 3.6. Field Emission Scanning Electron Microscope (FESEM) Ultimate Tensile Strength Fracture Surface

**Figure 16:**
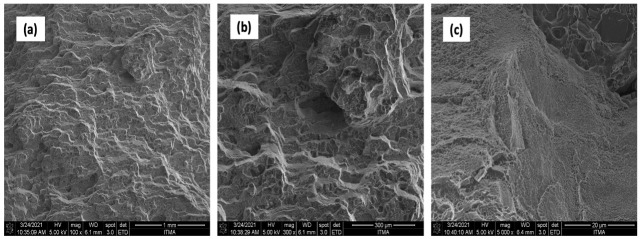
AA7075 AS (**a**) 100× (**b**) 300× (**c**) 5000×.

The FESEM analysis showed that hot extruded specimens exhibited no voids or cracks. The density test confirmed this FESEM finding; samples that underwent extrusion followed by heat treatment showed a density of 2.89 g/cm^3^. This value was higher than that of the AR samples. The density test strongly indicated possible porosities and a strong correlation with the porosity of the material. In comparison, hot extruded samples had less particle agglomeration and smaller grain sizes in the AFM sample profile (Table 11). Grain sizes were congruent with the results of AR AA7075. Figure 16 shows the boundaries of hot extruded samples for the AR sample.

**Figure 17:**
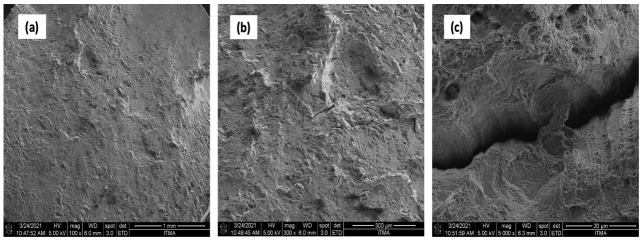
AA7075 chips (**a**) 100× (**b**) 300× (**c**) 5000×.

Uniformly distributed dimples and small cracks could be seen on the surface of the sample AA7075 chips, as shown in Figure 17. At 550 °Ċ, 3 h, clear tears and micro cracks were observed on the fracture.

**Figure 18:**
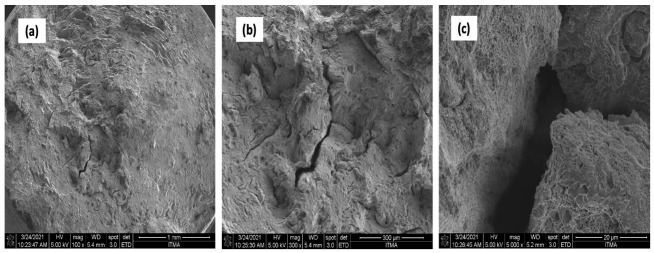
AA7075 1% vol ZrO_2_ (**a**) 100× (**b**) 300× (**c**) 5000×.

Additionally, Figure 18 shows the presence of uniformly distributed dimples and small cracks on the fracture surface of the sample reinforced with 1 vol% ZrO_2_. Tears and micro-cracks appeared at 550 °C and 3 h, and cleavage planes decreased obviously. An outer topography was visible (Figure 18a). The poor bonding between the chips showed a ridge instead of equiaxed dimples. Voids and pores were seen on the chip boundaries that resulted in oxidation during the fabrication process.

**Figure 19:**
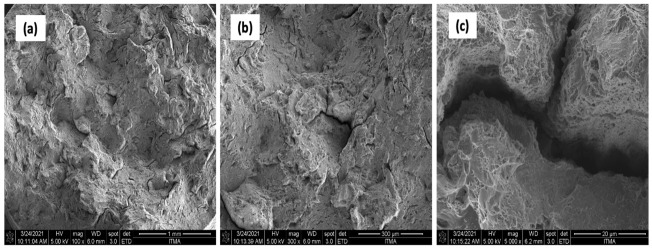
AA7075 1% vol ZrO_2_/HT (**a**) 100× (**b**) 300× (**c**) 5000×.

UTS and microhardness were enhanced in heat treatment, leading to a lack of voids and cracks in the sample’s microstructure. Figure 19 shows the microstructure of heat-treated samples. Nanomaterial reinforcement reduced the cracks (Figure 19b) and hence, the ultimate tensile strength and microhardness.

### 3.7. Atomic Force Microscope Analysis (AFM)

The microstructure was changed altered during the extrusion process, depending on the ZrO_2_ particle content in aluminium chips. The grains were less organised. When the volume fraction of particles was increased, the surface’s appearance was significantly increased after the hot extrusion forming process. All of the obtained grains, polycrystalline, and structure appeared to depend on the materials’ film thickness, and the lateral effective grains distributions are presented in Table 11 and Table 12. The most presented lateral decreased scales in the film thickness are 0.49 µm to o.286 µm after extrusion and from 0.488 to 0.433 after ECAP. The arithmetic roughness (Ra) for all samples decreased from 13.849, 9.879, and 16.279 (nm) of AA7075 chips, 1% vol of ZrO_2_ before and after heat treatment, respectively. The root means square (Rq) also was reported as 17.43 nm, 12.558 nm, and 19.427 nm for AA7075 chips, 1% vol of ZrO_2_ produced by extrusion and 1% vol of ZrO_2_ produced by extrusion followed by ECAP, respectively. It was confirmed that the aluminium composite properties were enhanced, with the grain size reduction of the investigated samples being related to the increases in the time spent and its growth.

Borblik et al. [19] proposed that the decreases in the size of sample resulted in decreases in all characteristics of the material surface. Luce et al. [20] reported the use of AFM to analyse sizes and other important characteristics for surface topography with ease in sample preparation for nano or microscales of the magnetic thin films.

## 4. Conclusions

This study focused on conducting a comprehensive investigation and computational analysis on the effects of preheating temperature (T), preheating time (t), and volume fraction (VF) of zirconium oxide on the mechanical properties and microstructure of the AA7075 composite manufactured by multiple processes. RSM revealed the main factor involved in achieving better UTS and microhardness was T and VF. On the other hand, t affected density. A decrease in VF increased the mechanical properties up to 1 vol% of oxide. With T at 550 °C, t at 1.58 h and ZrO_2_ at 1 vol%, the maximum hardness of 95 HV, a density of 2.85 g/cm^3^ and tensile strength of 487 MPa were obtained. All the factors T, t, and VF are considered as important factors that affect the nanocomposite. UTS and microhardness were sensitive to heat treatment, as an increase of 22% and 29%, respectively, was observed at high temperatures, efficiently consolidating the material. Density and microhardness of 2.9 g/cm^3^ and 140 HV, respectively, were obtained after ECAP followed by the heat treatment process. Compression pressure and extrusion stress produced in these stages were sufficient to eliminate voids that increased mechanical properties but were incapable of improving chip welding.

## Figures and Tables

**Table 1:** Aluminium AA7075 chips’ sizes.

Dimension	Chip 1	Chip 2	Chip 3	Chip 4
Width (µm)	67.7	58	44	56.5
Thickness (mm)	0.049	0.074	0.067	0.063
Length (mm)	3.42	3.1	3.5	3.34

**Table 2:** AA7075 aluminium chemical composition in wt.%.

Element	Percent (wt.%)	Atomic Mass (u)
Si	0.1	27.97
Fe	0.19	55.84
Cu	1.53	63.54
Mg	2.55	24.3
Zn	5.89	65.38
Mn	0.07	54.93
Cr	0.18	51.99
Ni	0.0058	58.69
Ti	0.024	47.86
Al	Bal	26.98

**Table 3:** The EDX analysis of the ZrO_2_ nano powder.

Reinforcement	Element	Weight%	Atomic%
ZrO_2_	O K	25.27	65.85
	Zr L	74.73	34.15
	Total	100	----

**Table 4:** The design scheme of the process parameters.

Factor Symbol	Parameter	Levels		
		Low (−1)	Centre (0)	High (+1)
T	Preheating temperature (°C)	450	500	550
t	Preheating time (hour)	1	2	3
VF	Volume fraction of zirconium oxide (%)	1	3	5

**Table 5:** Factors used in conducting hot extrusion.

Parameter	Value/Type
Shape of the die	Round
Ratio used in extrusion, R	5.4
Diameter of the billet, Ø (mm)	30
Speed during extrusion, s (mm/s)	1
Container temp, Tcont (°C)	300
Die temp., Tdie (°C)	300

**Table 6:** The analysis of variance of UTS by RSM.

Source	DF	Adj SS	Adj MS	F-Value	*p*-Value	Effect
Model	10	20,745.4	2074.54	29.74	0.000	Significant
Blocks	1	22.1	22.08	0.32	0.581	
Linear	3	8308.4	2769.47	39.70	0.000	Significant
Temp. (°C)	1	5307.2	5307.25	76.08	0.000	Significant
Time (hour)	1	91.1	91.08	1.31	0.269	Not significant
ZrO (Vol%)	1	2910.1	2910.08	41.72	0.000	Significant
Square	3	3098.9	1032.95	14.81	0.000	Significant
Temp. (°C) × Temp. (°C)	1	4.0	4.02	0.06	0.813	
Time (hour) × Time (hour)	1	67.1	67.15	0.96	0.340	
ZrO (Vol%) × ZrO (Vol%)	1	2278.2	2278.17	32.66	0.000	
2-Way Interaction	3	8281.5	2760.49	39.57	0.000	Significant
Temp. (°C) × Time (hour)	1	67.4	67.45	0.97	0.339	
Temp. (°C) × ZrO (Vol%)	1	5184.4	5184.36	74.32	0.000	
Time (hour) × ZrO (Vol%)	1	3029.7	3029.68	43.43	0.000	
Error	17	1185.8	69.75			
Lack-of-Fit	5	417.8	83.57	1.31	0.325	Not significant
Pure Error	12	768.0	64.00			
Total	27	21,931.2				

**Table 7:** The analysis of variance of hardness by full factorial.

Source	DF	Adj SS	Adj MS	F-Value	*p*-Value	Effect
Model	8	1418.66	177.333	44.24	0.000	Significant
Linear	3	961.29	320.429	79.95	0.000	Significant
Temp. (°C)	1	574.79	574.791	143.41	0.000	Significant
Time (hour)	1	11.37	11.370	2.84	0.123	Not significant
ZrO (Vol%)	1	375.13	375.127	93.59	0.000	Significant
2-Way Interactions	3	160.75	53.583	13.37	0.001	Significant
Temp. (°C) × Time (hour)	1	0.04	0.042	0.01	0.921	
Temp. (°C) × ZrO (Vol%)	1	23.61	23.606	5.89	0.036	
Time (hour) × ZrO (Vol%)	1	137.10	137.100	34.21	0.000	
3-Way Interactions	1	288.29	288.293	71.93	0.000	Significant
Temp. (°C) × Time (hour) × ZrO (Vol%)	1	288.29	288.293	71.93	0.000	
Curvature	1	8.33	8.332	2.08	0.180	Not significant
Error	10	40.08	4.008			
Total	18	1458.74				

**Table 8:** The analysis of variance of density by full factorial.

Source	DF	Adj SS	Adj MS	F-Value	*p*-Value	Effect
Model	6	0.027477	0.004580	169.94	0.000	Significant
Linear	3	0.017620	0.005873	217.95	0.000	Significant
Temp. (°C)	1	0.007801	0.007801	289.50	0.000	Significant
Time (hour)	1	0.003156	0.003156	117.10	0.000	Significant
ZrO (Vol%)	1	0.006663	0.006663	247.24	0.000	Significant
2-Way Interactions	2	0.008948	0.004474	166.02	0.000	Significant
Temp. (°C) × Time (hour)	1	0.000644	0.000644	23.89	0.000	
Temp. (°C) × ZrO (Vol%)	1	0.008304	0.008304	308.14	0.000	
Error	12	0.000323	0.000027			Significant
Lack-of-Fit	2	0.000110	0.000055	2.57	0.126	Not significant
Pure Error	10	0.000214	0.000021			
Total	18	0.027801				

**Table 9:** The confirmation test results.

CT	Temp	Time	ZrO_2_	Experimental			Predicted		
				UTS	Hardness	Density	UTS	Hardness	Density
1	550	1.58	1	487.78	97	2.75	490.23	102.56	2.87
2	542	1	1	489.88	103	2.8	488.55	102.42	2.86
3	550	1.58	0.8	469	95.2	2.81	500.01	99.90	2.87

**Table 10:** RSM prediction error.

Prediction Error	Error %		
	UTS	Hardness	Density
CT 1	0.50	5.73	4.44
CT 2	0.27	0.57	2.12
CT 3	6.61	4.94	2.22

**Table 11:** AFM topography images of recycled samples.

Samples	Topography	Surfaces Roughness
Chips AA7075	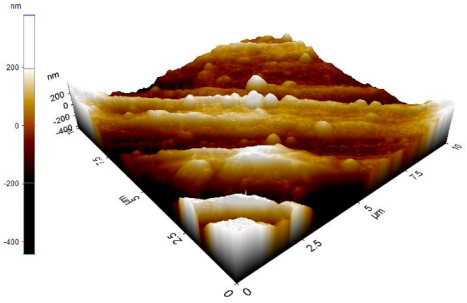	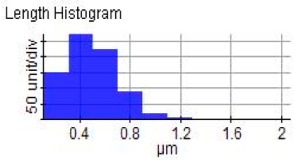
AA7075 1 vol% ZrO_2_ Hot Extrusion	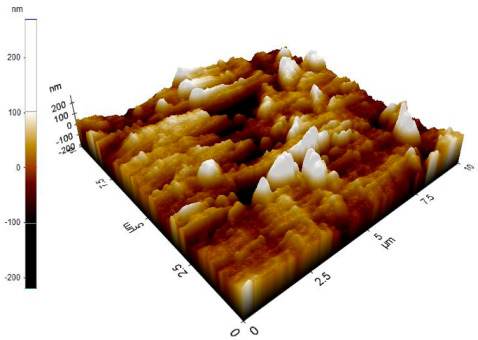	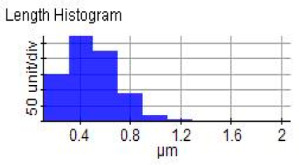
AA7075 1 vol% ZrO_2_ Hot Extrusion/HT	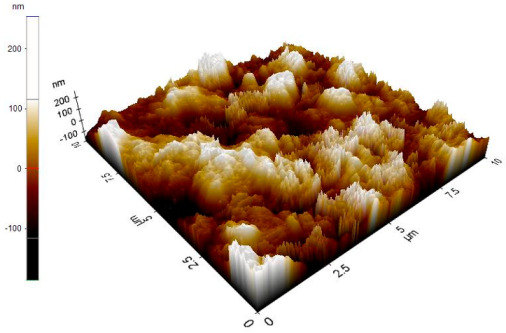	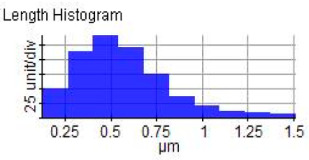
AA7075 1 vol% ZrO_2_ Hot Extrusion/ECAP	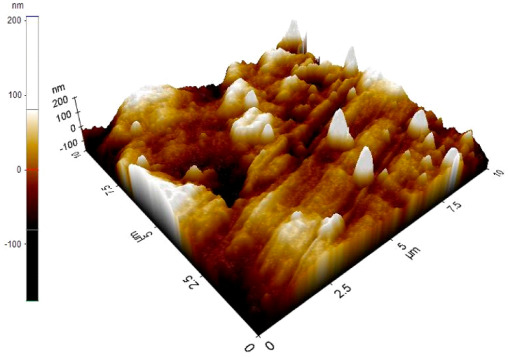	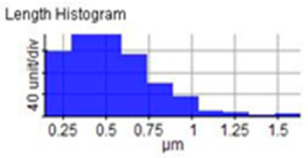
AA7075 1 vol% ZrO_2_ Hot Extrusion/ECAP/HT	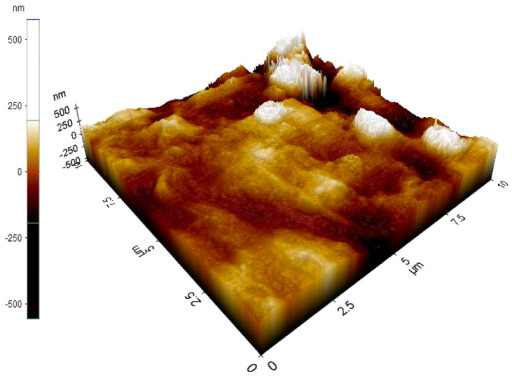	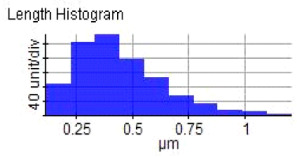

**Table 12:** AFM mean grain size and surfaces roughness.

Samples	Mean Grain Size (µm)	Ra (nm)	Rq (nm)
Chips AA7075	0.490	13.84	17.43
AA7075 1% vol ZrO_2_ Hot Extrusion	0.486	9.87	12.55
AA7075 1% vol ZrO_2_ Hot Extrusion/HT	0.488	16.27	20.63
AA7075 1% vol ZrO_2_ Hot Extrusion/ECAP	0.478	15.11	19.42
AA7075 1% vol ZrO_2_ Hot Extrusion/ECAP/HT	0.433	28.21	34.07

## Data Availability

Not applicable.
